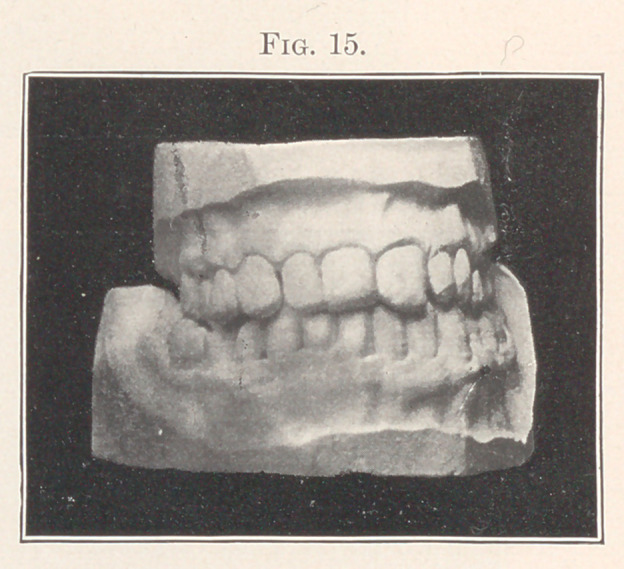# A Report on Orthodontia

**Published:** 1904-11

**Authors:** Lawrence W. Baker

**Affiliations:** Boston, Mass.


### A REPORT ON ORTHODONTIA.[1]

BY LAWRENCE W. BAKER, D.M.D., BOSTON, MASS.

At the request of our president, Dr. Stanley, I have prepared for you to-night a report on the subject of Orthodontia. This report will consist of the explanation of some lantern-slides illustrating a series of cases that I have treated by means of the expansion arch appliance, perhaps better known to you as the Angle appliance.

Owing to the full programme, I shall endeavor to be as brief as possible.

The first slide that I am to have thrown upon the screen (Fig. 1) represents a type of normal occlusion which is familiar to you all. The more of this work I do the more I see to study and admire in this wonderful piece of nature's work, and I trust that by again bringing this picture to your attention you may also profit by it. It is the model that I have attempted to approach in the treatment of the various cases that we are to consider. I only wish that this picture could be permanently placed upon the screen, so that it could be compared with each and every case we are to discuss; for I fully realize that in order to obtain permanent results in this work the establishment of normal occlusion is essential.

It is essential simply for this one reason—that when the teeth are in a state of normal occlusion there is produced an *equilibrium of the occlusal forces* which can be obtained in no other possible arrangement of the occlusal planes. We should study, plan, and work to obtain this balance, for with it the teeth are placed in their most *retentive positions,* and there is the least possible chance of the

---

[1] Read before the Harvard Odontological Society, April 28, 1904.

arches returning to their malformed condition when once the tissue about the teeth becomes normal.

Perhaps by classifying these cases into several groups I may be able to explain them more clearly. I have adopted the Angle classification which has done so much for the advancement of this science by giving us a definite basis to work upon.

The first two cases have this one feature in common,—that the first molars occlude normally mesio-distally, placing them in the first group. Cases III., IV., and V. are characterized by a distal displacement of the first molar; therefore they belong to the second group. In Case VI. the occlusion has been so mutilated by extraction that I have been forced to put it in an out-of-class group.

Since this classification is based upon the normal mesio-distal relations of the first molars, let us consider our model and see what their normal relations are. We find that the lower first molar is one occlusal plane in advance of the upper one, allowing the point of the upper antro-buccal cusp to fall into the buccal fissure of the lower first molar, as shown by the lines marked upon these landmarks.

Case I.—Fig. 2 shows us two side views of a case before treatment. The relations of our molar landmarks are normal, placing this case in the first class, according to our Angle classification. The reason for attempting correction of this case is evidently to improve the facial and dental expression.

Fig. 3 shows the results of the attempt at copying our model, normal occlusion. This change has given us a marked improvement in appearance and in utility, and has also greatly minimized the danger from dental caries, all resulting from the fact that the balance of the occlusal forces has been established.

Case II.—Fig. 4 illustrates similar views of a case having this one occlusal characteristic in common with the preceding,—the molar landmarks are normal mesio-distally, although the cases differ widely in other respects.

Fig. 5 shows the results obtained. I think you will all readily appreciate that the occlusal balance was reached, when I tell you that, after correction, I depended largely upon the occlusal force for retention. This normal locking of the teeth not only held what I had gained, but greatly improved upon my work.

Case III.—Fig. 6. We notice at once that the character of this

Fig. 1.

Fig. 2.

Fig. 3.

Fig. 4.

Fig. 5.

Fig. 6.

Fig. 7.

case is entirely different from that of the two preceding ones. It belongs to a different class,—to Group II.,—because of the distal displacement of the lower arch.

Formerly this deformity was supposed to be confined to a protrusion of the upper arch alone, but since the importance of the relations at normal occlusion to this work was realized, it has been noted that this deformity is caused by a distal displacement of the lower arch, as well as to a slight protrusion of the upper incisors. The deformity of one arch unfortunately exaggerates that of the other, producing a marked facial disfigurement, which can be easily imagined by studying the models before treatment. Fig. 7 shows the results obtained. The protrusion of the upper incisors has been reduced, and at the same time the receding lower jaw has been brought forward to its intended position, producing a great improvement in the balance of the facial lines; which goes to show that the relations between facial and dental harmony are closely associated. This result was obtained by the use of the inter-maxillary elastics, first used with marked success by my father, Dr. H. A. Baker.

Case IV.—This I consider the most interesting case that I am to present this evening. It is one of those rare cases of distal occlusion in which the bite was jumped entirely unaided. To understand the changes that took place, close attention must be paid to the illustrations and description.

The apparent deformity is confined to the anterior part of the superior arch (see Fig. 9), but on consulting our occlusal landmarks in Fig. 8 it is seen that the lower arch is in distal occlusion, which places this case in the same general class with the preceding one. However, in that case the incisors *protruded,* while in this case they *retrude,* which fact places them in different subclasses of this second division.

Fig. 9 shows the occlusal aspect of the two arches before treatment. Note the crowded condition of the upper incisors, while the lower arch is perfectly regular.

Fig. 10 presents the same view of the corrected models. The only change noted is the correction of the upper arch, the lower arch remaining unaltered; in fact, no force whatever was applied to it during the progress of the work.

The next figure, 11, shows the effect of this change upon the occlusion. See that the receding lower arch has come forward to its correct position; the contraction of the anterior part of the upper arch having been the cause of its distal displacement. When the upper arch was made normal the lower arch simply slid forward to its proper place,—another case in which we see that as soon as nature was given a chance she re-established the occlusal balance.

Case V.—It is with great hesitancy that I present this case, for two reasons: in the first place, I fear that you will doubt that there could be, due to the teeth, such a malformation of the jaws; and again, because I am unable to give you the finished result, the case being still under treatment.

Fig. 12 shows that the lower arch is not only in distal occlusion, but is doubly so; that is, instead of being one plane distal, it is distal two planes, causing a marked facial deformity.

By way of history, I should like to state that when the child came to me the lower sixth-year molars were beyond saving. At the proper time I had the bits of roots removed, allowing the twelfth-year molars to come forward, with the result that you see here.

In undertaking this case I should have hesitated at the responsibility had I not known something of the possibilities of the intermaxillary elastics.

The next illustration, Fig. 13, shows the study models as the case is. To get the exact relations of the two jaws I had the patient bite in wax and placed the models together accordingly. I am of the opinion that later, when the occlusion settles, a permanent benefit will result.

In this case many would have considered extraction in the upper arch necessary. I believe that by keeping the upper arch intact and bringing this receding lower jaw forward the facial lines will be placed in much better balance; for instead of weakening the lower part of the face, by moulding it to the weak receding lower jaw, it was strengthened by bringing the chin forward to harmonize with the general facial contour.

Case VI.—Figs. 14 and 15. After considering the preceding case the one at hand will appear very simple. It is one of those cases in which there has been such wholesale extraction that I considered it impossible to obtain the normal state of affairs, so worked to get the best abnormal occlusion I could. For this reason we might consider it an "out-of-class" case, and I have placed it at the end of the report.

It is similar to most cases in which the arches have been mutilated by extraction, in that the deformity was progressive. In fact,

Fig. 8.

Fig. 9.

Fig. 10.

Fig. 11.

Fig. 12.

Fig. 13.

Fig. 14.

Fig. 15.

recently conditions had changed for the worse so rapidly that an increasing facial deformity was resulting, as might be expected from studying the models before treatment. The patient being a middle-aged woman, I could not anticipate an ideal result.

In bringing the retruding superior teeth over the lower ones spaces were necessarily formed. This condition was overcome by carrying the poorly spaced teeth towards the median line, converting the spaces into one large one between the cuspid and bicuspid and supplying this space with what might be termed a retaining bridge.

From the cases that we have briefly considered some may get the idea that I am an extremist regarding the non-extraction of teeth in the practice of Orthodontia. While my observations are that the use of the forceps has caused more harm than good, still we do find cases which have been previously mutilated by extraction, where an occlusal balance is impossible, and in which by resorting to judicious extraction, to balance planes previously lost, we may improve conditions by getting the best abnormal occlusion possible. Or, again, we find a limited number of cases where the lost planes can be successfully restored artificially.

In cases where the teeth are all present many mouths are ruined by the carrying out of the erroneous idea that extraction simplifies matters, whereas, as a rule, the case is usually made more difficult to correct and to retain.

## Figures and Tables

**Fig. 1. f1:**
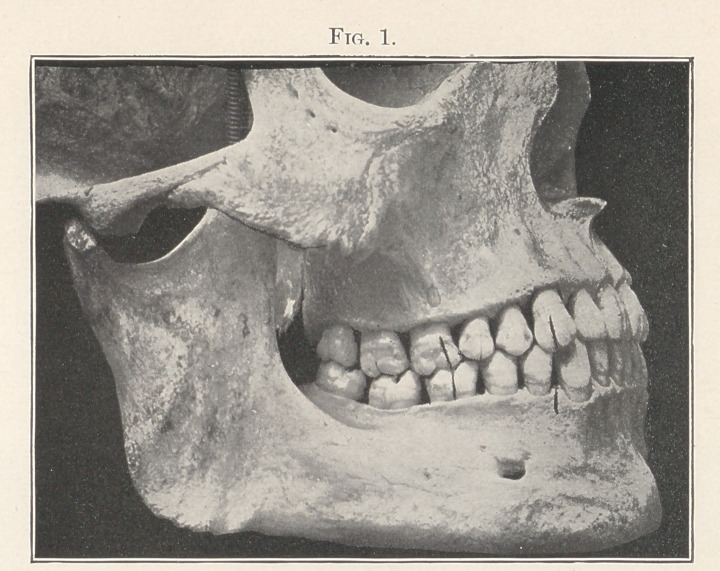


**Fig. 2. f2:**
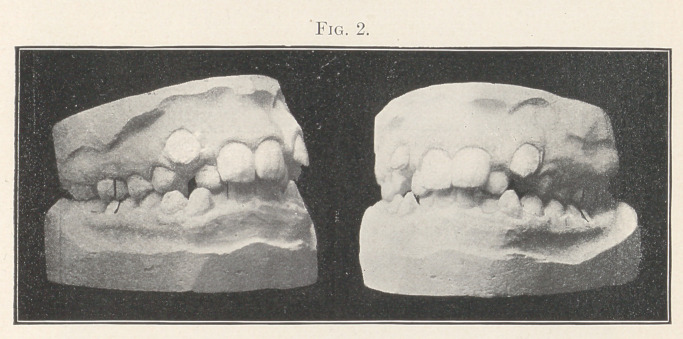


**Fig. 3. f3:**
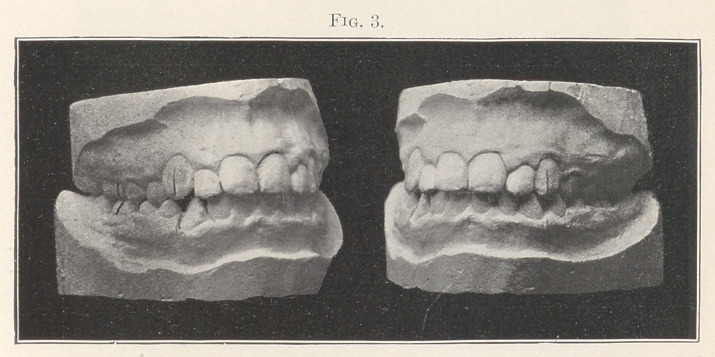


**Fig. 4. f4:**
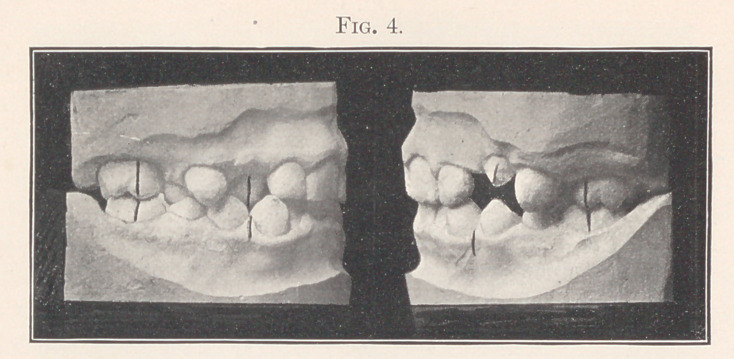


**Fig. 5. f5:**
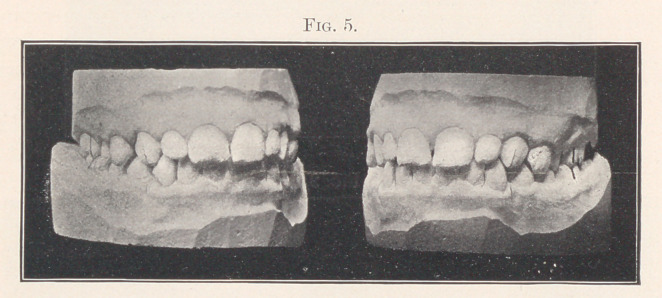


**Fig. 6. f6:**
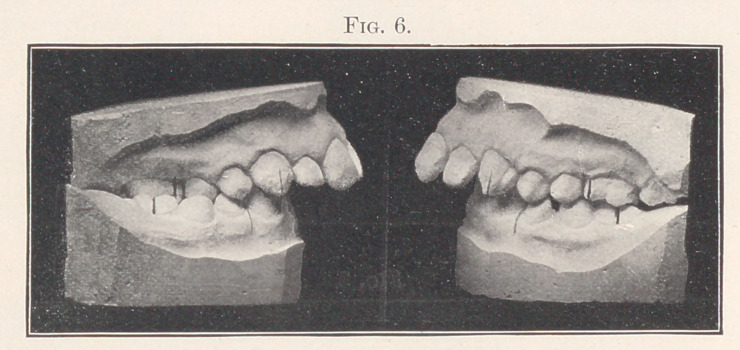


**Fig. 7. f7:**
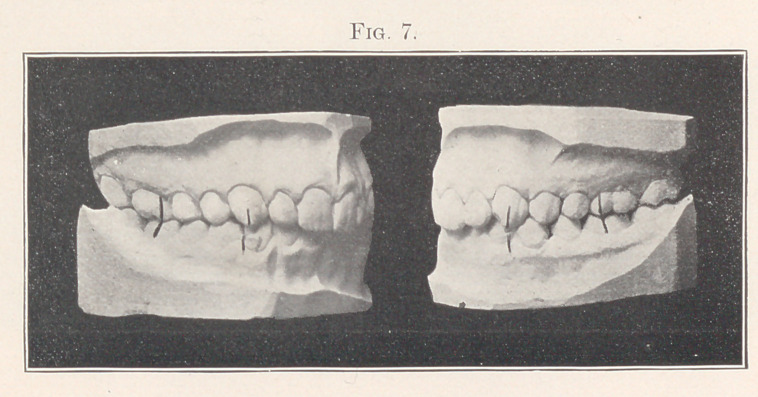


**Fig. 8. f8:**
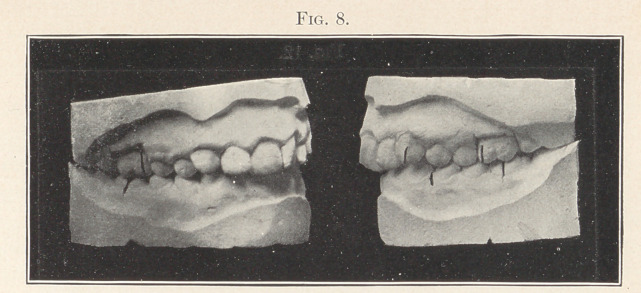


**Fig. 9. f9:**
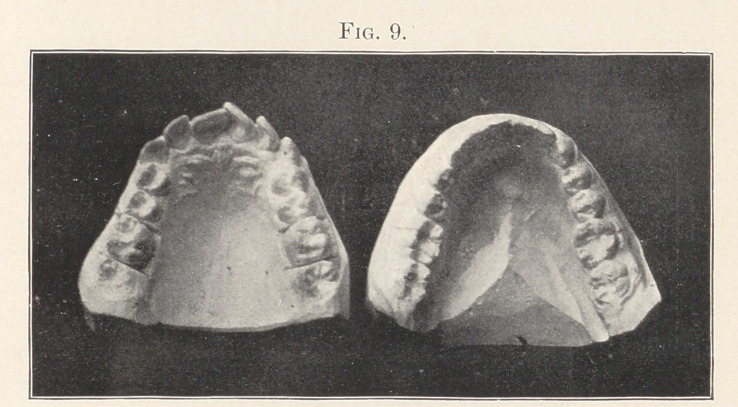


**Fig. 10. f10:**
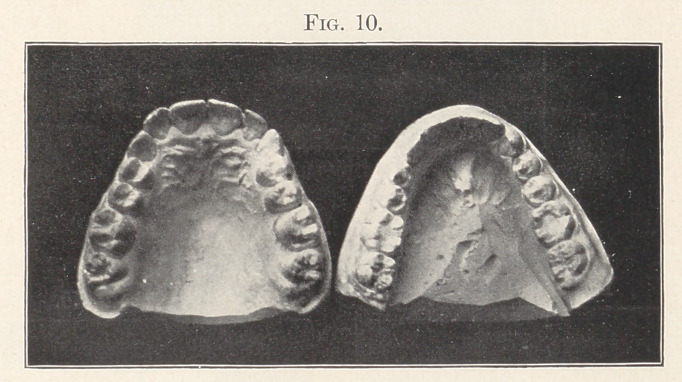


**Fig. 11. f11:**
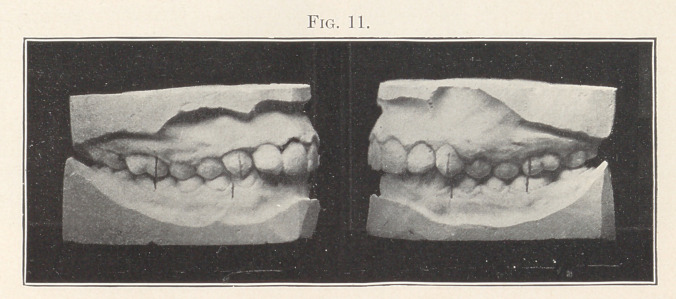


**Fig. 12. f12:**
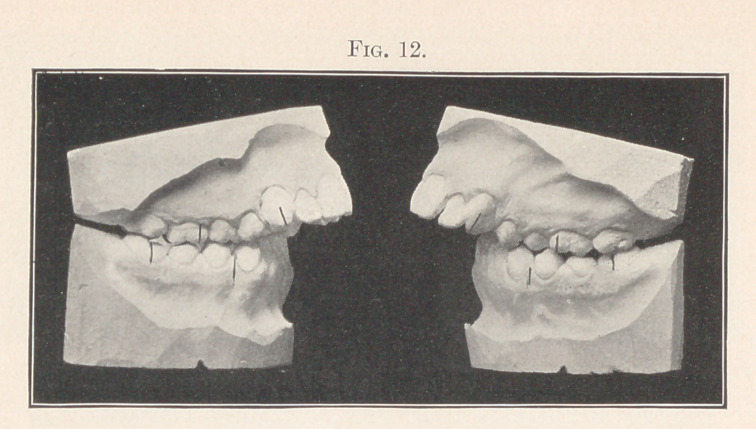


**Fig. 13. f13:**
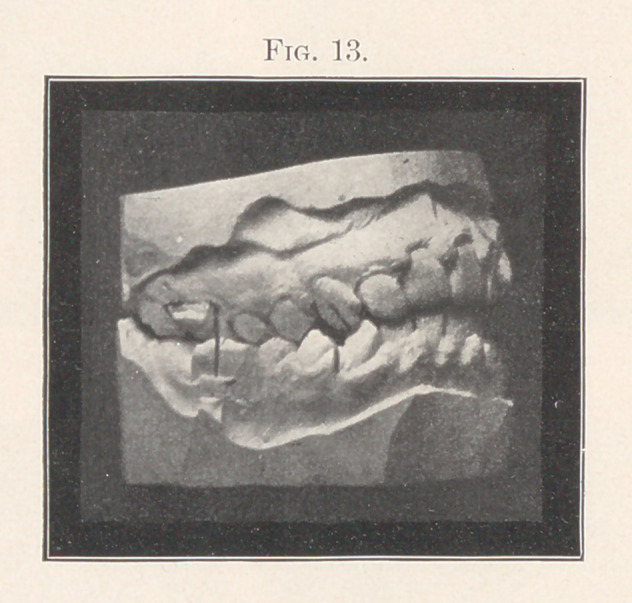


**Fig. 14. f14:**
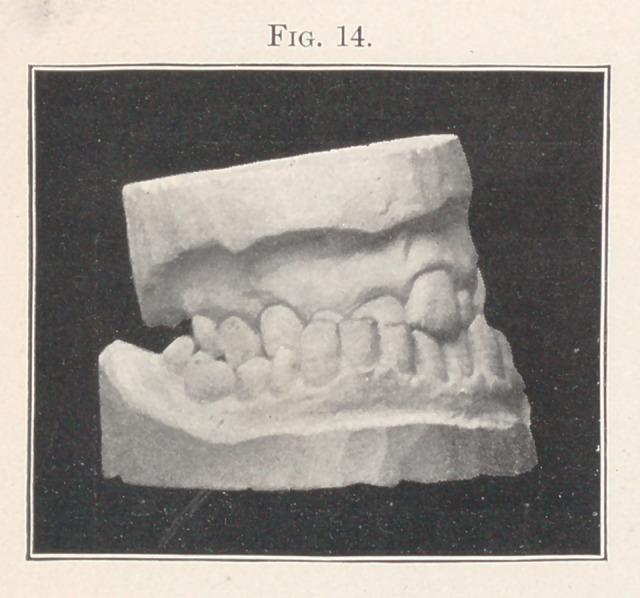


**Fig. 15. f15:**